# Promoting Healthy Relationships in Foster Care—“If I Had Seen What a Healthy Relationship Looks Like, that Would Have Changed My Perspective”

**DOI:** 10.1177/10778012231199106

**Published:** 2023-09-07

**Authors:** Barbara Ball, Sharon Hoefer, Xiao Ding, Lalaine Sevillano, Monica Faulkner

**Affiliations:** 1 Texas Institute for Child and Family Wellbeing, Steve Hicks School of Social Work, 12330 The University of Texas at Austin, Austin, TX, USA

**Keywords:** foster care, emerging adulthood, intimate partner violence, violence prevention, qualitative research

## Abstract

High levels of intimate partner violence among youth and young adults with history in foster care can perpetuate the cycle of violence and abuse. It is therefore important to understand how the experience of growing up in foster care impacts youths’ understanding and formation of intimate relationships. This qualitative study centered the perspectives of young adults and investigated what they learned about relationships through interactions with foster caregivers and child welfare professionals. We conducted semi-structured interviews with 27 young adults and used Consensual Qualitative Research methods to identify critical experiences in foster care and formulate strategies for promoting healthy relationships.

## Introduction

Romantic relationships with high levels of intimacy, support, affection, and satisfaction, contribute to social emotional wellbeing and a sense of self-worth and competence (Collins et al., 2009). Indeed young adults with a history in foster care report that having a romantic partner is the social connection that most significantly increases their perception of social support and wellbeing (Zinn et al., 2017). However, high levels of intimate partner violence (IPV) among youth and young adults with history in foster care (Herrman et al., 2016) can perpetuate the generational cycle of violence and abuse. A majority of youth in the foster care system have experienced multiple forms of victimization across social contexts (Cyr et al., 2012) that increase their risk for IPV. The intervention of the child welfare system, the removal from their birth family, relationships with foster caregivers and child welfare professionals, and eventually the transition out of the child welfare system pose a unique set of opportunities and challenges. It is therefore important to understand how the experience of growing up in foster care impacts youths’ understanding and formation of intimate relationships. The present study centered the perspectives of young adults and investigated what they learned about relationships through interactions with foster caregivers and child welfare professionals and how their understanding of themselves and intimate relationships evolved in emerging adulthood.

### Risk and Protective Factors for IPV in the Lives of Youth in Foster Care

According to the Adoption and Foster Care Analysis and Reporting System (AFCARS), there were 391,098 children in foster care at the end of Fiscal Year 2021, among them 110,230 youth ages 13 and older (DHHS, 2022). Foster care, also known as out-of-home care for children who have been removed from their families, includes relative and non-relative foster family homes, pre-adoptive homes, and congregate care settings such as shelters, group homes and residential treatment centers. Black youth (Cénat et al., 2021) and lesbian, gay, bisexual, transgender, questioning, and queer (LGBTQ+) youth (Baams et al., 2019) are overrepresented among youth in foster care, stay in foster care longer, and are more likely to live in congregate care than their peers.

Youth in foster care experience significantly elevated rates of IPV victimization, which can include physical violence, sexual violence, stalking, or psychological harm by a current or former partner. Herrman et al. (2016) found that 36% of youth in out-of-home care who were dating had experienced physical and sexual IPV in the past 12 months compared to 12% of youth nationally (Basile et al., 2020). When emotional and verbal IPV were included, several studies demonstrated that between 40% and 90% of youth in out-of-home care experienced some form of victimization in the past 12 months (Manseau et al., 2008; Taussig & Garrido, 2017).

IPV victimization in adolescence is a risk factor for continuing IPV in adulthood (Exner-Cortens et al., 2017) and approximately 21% of foster care alumni reported some type of physical or sexual IPV at age 23/24 (Katz et al., 2020). Consequences of IPV victimization include missed educational and career opportunities, housing and economic instability, negative health and mental health outcomes, injury, and death (Niolon et al., 2017). IPV also impacts sexual and reproductive health. Reproductive coercion, comprised of behaviors such as birth control sabotage, pregnancy coercion, and condom manipulation, is associated with sexually transmitted infections and unintended pregnancies (Miller et al., 2014). The high prevalence of IPV and the associated consequences call for the development of effective prevention and intervention strategies that target specific risk and protective factors among youth in foster care. Existing research points to intersecting risk factors at the individual, interpersonal, and systems levels that include persistent trauma symptoms, placement instability and lack of connections with caregivers, and restrictive practices and policies in the child welfare system.

#### Persistent Trauma Symptoms and Negative Self-Concept

Children and youth who enter the child welfare system typically have a history of inconsistent, neglectful, or abusive relationships. In addition, their experiences might include peer, school, and community violence and immigration related violence all of which are frequently underreported in the child welfare system (Cyr et al., 2012; Loomis et al., 2020). A majority of children in the child welfare system have experienced poly-victimization (Cyr et al., 2012), which is highly predictive of experiencing complex trauma symptoms (Finkelhor et al., 2015) that include an impairment in core capacities for affect regulation, behavioral regulation, cognitive and executive functioning, self-concept, and interpersonal relatedness (Cook et al., 2005). Several studies have found an association between persistent trauma symptoms and increased risk for chronic victimization and IPV in adolescence and emerging adulthood (Taussig & Garrido, 2017; Wekerle et al., 2009). Other studies have explained the link between childhood exposure to violence and IPV perpetration and victimization in adulthood from a social learning perspective. Children who experience violence and abuse across multiple social contexts, learn and internalize abusive relationship models, accept coercive and power-based norms as ways for regulating conflict, and may seek peers and partners with similar experiences, thus continuing the cycle of abuse (Ehrensaft et al., 2003). Exposure to violence also impacts the formation of a child's self-concept. Childhood sexual abuse, in particular, is associated with negative self-perceptions, stigma, shame, self-blame, and feelings of powerlessness (Hébert et al., 2017). Taken together these negative self-perceptions may make it difficult for youth to communicate assertively, set sexual boundaries, or negotiate condom use and birth control (Thompson et al., 2017), and increase the risk of experiencing severe violence (Collin-Vézina et al., 2006). Histories of child sexual abuse are common among youth in foster care (Ahrens et al., 2012) and are associated with transactional sex and sexual victimization later in life.

#### Placement History and Relationship With Foster Caregivers

The placement of children and youth in foster care, a formal intervention, is intended to provide opportunities for healing and growth. However, older youth (ages 14–17) experience high rates of placement instability, with 40% having four or more placements during their most recent stay in care (Sattler et al., 2018). Older youth are at a particularly high risk of congregate care placement (Covington et al., 2022) where they report having less social and emotional support than their peers in family-like settings and lack trusting relationships with the adults who care for them (Okpych et al., 2018). Although foster care is intended as a safe space, reports of neglect and abuse in care are alarmingly high. Using self-reports, one study with youth who were emancipating from foster care found that about one third of these youth had experienced neglect by a foster caregiver, about one quarter had experienced physical abuse by a foster caregiver, and on fifth had experienced sexual abuse with an unknown perpetrator (Katz et al., 2017).

Placement instability and neglect in care are associated with IPV involvement in young adulthood (age 23/24 years) (Katz et al., 2020). Conversely, placement stability (Jonson-Reid et al., 2007), positive parenting practices and closeness to caregivers (Garrido & Taussig, 2013) have been identified as protective factors against IPV. Potter and Font (2019) showed that caregiver closeness irrespective of placement type reduced the likelihood of youth engaging in sexual behaviors such as unprotected intercourse and sexual activity with new partners. Garrido and Taussig (2013) hypothesized that positive parenting practices may provide a template for healthy relationships and a safe space to share experiences and build skills, such as emotion regulation.

However, several studies have demonstrated that child welfare professionals and foster caregivers do not consistently talk with youth in their care about sexuality and relationships (Albertson et al., 2018; Harmon-Darrow et al., 2020; Serrano et al., 2018). Caregivers and child welfare professionals cited discomfort talking with youth of different genders and sexual orientations; lack of training and guidance; and religious and personal beliefs that might differ from the youths’ beliefs of what constitutes normal and acceptable behavior. Lesbian, gay, bisexual, transgender, and queer/questioning (LGBTQ) youth, overrepresented in the child welfare system, face special barriers with regard to adequate placements and placement stability, caregiver support, and exploration of sexual identity and gender expression (Baams et al., 2019).

#### Normalcy in the Child Welfare System

Another well-documented challenge for youth in the child welfare system are policies and practices that limit “normal” age-appropriate activities, such as learning to drive, socializing with peers, and dating, due to safety and liability concerns. These policies constrain adolescents’ developmental needs to explore their identity and relationships, increase their independence, and take healthy risks (Simmons-Horton, 2017). The Preventing Sex Trafficking and Strengthening Families Act of 2014 sought to promote normalcy by introducing the “reasonable and prudent parent standard,” which allows foster caregivers to make day-to-day decisions about a youth's participation in age-appropriate social activities. The implementation of normalcy provisions is, however, complex and caregivers report challenges with balancing adequate monitoring and providing youth opportunities to explore relationships and develop skills and independence; this is especially true for youth who have experienced significant trauma that makes them vulnerable for dating and sexual victimization (Albertson et al., 2018).

### Purpose of the Present Study

In summary, existing research on the romantic relationships of youth and young adults with lived experience in the foster care system demonstrates the high prevalence of IPV and the accumulation of risk factors across different contexts and development. There is some indication that stable and close relationships with foster caregivers, supportive adults, and child welfare professionals may have a protective function and provide opportunities for youth to observe and practice social skills and become aware of choices they can make for healthier and safer relationships (Wekerle et al., 2009). However, extant research also documents the hurdles in the child welfare system and among caregivers with regard to supporting youth as they explore dating and sexual relationships.

The present study addresses a gap in research by centering the perspectives of young adults. We focused on the following questions: (a) What do youth in foster care learn from caregivers (foster, adoptive, kinship caregivers) about healthy, unhealthy, and abusive relationships? How do their interactions with caregivers impact their understanding of themselves and relationships? (b) How do youth describe their relationships with peers and intimate partners? (c) How does their understanding of themselves and their relationships evolve over time? What experiences allow them to grow and develop healthy relationships in emerging adulthood? We conducted individual, semi-structured interviews (approximately 60–90 minutes) with a diverse group of 27 young adults, ages 18–30, who had lived in foster care as adolescents and used Consensual Qualitative Research methods (Hill et al., 1997, 2005) to generate a deeper understanding of individual life trajectories, identify critical experiences across individuals, and identify strategies for promoting healthy relationships among youth in care.

## Method

### Participants

Participants were recruited through word of mouth, foster care alumni groups, referrals from service providers, and foster care liaisons at college campuses. Interested individuals were eligible for the study if they (a) were at least 18 years old; (b) had spent time in foster care as adolescents (between the ages of 13 and 18); and (c) appeared to have no mental health, substance use, or developmental impediments that would prevent them from completing an individual interview. Eligible participants were contacted by the principal investigator to obtain written consent. Interviews were scheduled at a convenient and private location in the community or via phone. A $50 gift card was provided for completion of the interview.

The sampling strategy resulted in a convenience sample of 27 young adults. Intensive outreach efforts to recruit male and transgender youth had limited results and recruitment ended when the research team determined that data collection reached the point of saturation. Participants’ ages ranged from 18 to 30 years (*M *= 23.48, *SD *= 2.90). The majority of participants (55.6%; *n *= 15) identified as Hispanic, 14.8% (*n *= 4) as Non-Hispanic Black, and 29.6% (*n *= 8) as Non-Hispanic White. The sample was 81.5% (*n *= 22) cisgender women, 14.8% (*n *= 4) cisgender men, and 3.7% transgender women (*n *= 1); 63% (*n *= 17) of the participants identified as heterosexual or straight and 37% (*n *= 10) identified as lesbian, gay, or bisexual. Ninety-three percent (*n *= 25) of our sample had a high school diploma or GED and 70.4% (*n *= 19) were enrolled in college at the time of the interview or had completed an undergraduate degree. All participants had spent some time in foster care during adolescence, however their experiences in care varied significantly by age of first removal, number of placements, and exit from the foster care system (Table 1).

**Table 1.** Self-Reported Placement History.

Placement history	Number of participants (*N* = 27)
Age at first removal	
0–10	8
11–15	14
16 and older	5
Number of placements	
1–2	10
3–5	10
10–14	2
15 or more	5
Exit from foster care	
Emancipated at age 18	21
Adopted	5
Reunified	1

### Interview Protocol

The interview consisted of a set of eight open-ended questions (Table 2) that were intended to gather consistent information across participants while allowing opportunities for extensive probing and deeper exploration of participants’ emerging understanding of themselves and their intimate relationships (Hill et al., 2005). For the purposes of this study, dating was defined a priori as going out, hanging out with someone, being romantically involved with someone, or “hooking up” sexually with someone, including relationships of any length. All interviews were completed by the principal investigator who has extensive experience in counseling with child welfare-involved youth and dating violence prevention and intervention. Interviews were conducted in English and typically lasted between 60 and 90 minutes. Seventeen interviews were conducted on the phone, eleven in person; no differences in the interview process were noted. Interviews were recorded, transcribed, and checked for accuracy; identifying information was redacted prior to data analysis.

**Table 2.** Set of Interview Questions.

Primary interview questions	Sample follow-up questions
1. How would you describe relationships in your birth family?	When and why did you enter the foster care system?
2. Who were the important people in your life as a teen?	What were your experiences like at school and with peers?
3. When did you first start dating and what was it like for you?	How did you meet your partner(s)?
4. How would you describe your relationships with foster caregivers and other important adults at that time?	How did they respond when you started dating someone? What did you learn from them about relationships?
5. What type of sexuality and relationship education did you participate in?	What did you learn from these programs?
6. What are your relationships like now, as a young adult?	How do you decide whether a relationship is good for you?
7. Looking back, what have you learned about yourself and relationships?	What (or who) was helpful along the way? What is still hard for you?
8. What do you wish you had known when you were a teen?	What would help teens in care learn how to navigate relationships?

### Research Team

The research team acknowledges that our social identities shape how we approach research, from recruiting and interviewing participants to analyzing and interpreting the data. “Positionality is the notion that personal values, views, and location in time and space influence how one understands the world” (Sánchez-Ayala, 2012, p. 118). We offer our positionality statements to reflect on the ways in which we locate ourselves in society and construct knowledge that reflects intersections of our identities, such as race and ethnicity, gender, socioeconomic status, and health and disability status. We are a team of able-bodied, cisgender women who represent diverse backgrounds and professional experiences. The first author, born and raised in Germany, experienced unhealthy dating relationships in emerging adulthood that ultimately led her to develop strategies for dating and sexual violence prevention. The second author is a white woman from Texas who has worked professionally with young people with lived experience in foster care and with survivors of violence. The third author identifies as a Chinese immigrant from a remote ethnic minority-dominant region, who now works as a researcher and clinical social worker with at-risk youth in school settings. The fourth author identifies as a Pilipinx American from a low socioeconomic status, and a person who holds advanced degrees. The last author identifies as a mixed-ethnicity Hispanic and White woman who holds advanced degrees and works on improving the foster care system.

Consistent with consensual qualitative research methods (Hill et al., 2005) the research team developed the data analysis in a collaborative manner. The primary analysis team consisted of three researchers who jointly developed the coding system, analyzed the data, and kept memos and audit trails to support the transparency of the analytic process. The researchers first completed their tasks independently and then discussed their findings in weekly meetings to reach consensus. A team member, who did not participate in the initial analysis, served as auditor and independently reviewed data and emerging concepts. The auditor's feedback was incorporated into the iterative analytic process.

Throughout the research process, from interviews to data analysis and interpretation, it was paramount to refrain from imposing the researchers’ biases of what constitutes a healthy, unhealthy, or abusive relationship on the participants. For example, Claire (cisgender woman, straight, 21) when reflecting on a relationship at age 13 years stated, it “was pretty good because I got to stay with his mom at their house for a while. I got to get away from my mom. Every now and then he’d call me derogatory words, but it never got to the point where there was violence or anything. So, I would consider it, for the most part, a somewhat healthy relationship.” While there may have been the potential for harm in this dating relationship, team members challenged each other to listen closely to the participant's choice of language and understand how she made meaning of her relationships and coped with adverse life circumstances.

### Data Analysis

Data analysis involved three steps. In the first step, research team members worked together to develop domains and segment interview data based on an initial review of 10 transcripts. Once consensus for domain coding was achieved, all transcripts were coded in Dedoose by pairs of researchers who met weekly to discuss and reconcile any differences. In the second step, the team developed core ideas for each domain which consisted of succinct statements that summarized the participants’ words. Core ideas were first developed for individual interview responses and then refined across interviews. In the third step, the cross-analysis of data, the team examined connections between core ideas across interviews and developed a domain structure that best reflected the participants’ experiences. The team made note how frequently domains and core ideas appeared, and whether they applied to a majority (more than half) or minority (less than half) of cases (Hill et al., 2005).

## Findings

Research participants seized on the opportunity to talk about sexuality and relationships because they felt that conversations about these topics had been absent during their formative years. Pseudonyms were assigned to protect the participants’ confidentiality. Carolina (cisgender woman, straight, 24) summed it up, “One of the reasons this whole study interested me was because I feel like people who go through foster care, it's really hard to be able to communicate and talk about relationships. I never had an example of what a healthy relationship looks like and I never had anybody talk to me about that.” The following sections detail our findings concerning what participants learned about relationships while in foster care, how they formed intimate relationships, and how they reflected on ongoing growth as young adults. We are exploring three core domains—relationship models, connections with caregivers and peers, and sense of self and self-worth—across these sections of analysis.

### Learning About Relationships Through Interactions With Foster Caregivers

Participants overwhelmingly talked about missed opportunities for learning about healthy relationships in their interactions with foster caregivers and child welfare professionals. They perceived the foster care system as both protective and controlling by limiting their opportunities to explore relationships and their identities. Relatedly, they noted that caregivers and child welfare professionals did not talk to them about relationships and sexuality, or only in a negative, risk-centered manner which left them feeling disconnected and stigmatized.

#### Focus on Risks—“Foster Care is a Protective Bubble”

When reflecting on their experiences in foster care, a majority of participants described the negative impact of a system that viewed dating and sexual relationships as risks, placed them in a “protective bubble,” and limited opportunities for exploration, learning from mistakes, and making decisions.

I know from where I came from there's a reason why they were so bent on making sure I wouldn’t repeat the life of my parents. They are doing so much to protect you that they are putting you in a bubble that in itself is counterproductive. Because protection itself should also allow that person to explore who they are, allow them to make mistakes. But they didn’t allow for exploration. And all these permissions to go over to people's houses limiting the stuff that you do as a normal teenager because it's in those situations that you start learning about who you are. (Jonathan, cisgender man, gay, 30)

Participants noted that any sexual health and relationship education they received through the child welfare system (e.g., preparation for adult living classes) was fear-based, highlighting the warning signs of dating violence rather than skills for healthy relationships. “It was more how to avoid domestic violence than how to have a healthy relationship. It was ‘What are the red flags that I have to avoid?’ Foster care did not give me a lot of information how to have a healthy sexual relationship or how to enjoy each other” (Angelina, cisgender woman, straight, 21).

The overall perception of dating as a risk, translated into restrictive placement rules. Many participants reported that they were not allowed to date in their placements. Among them was Gabriela (cisgender woman, straight, 27) who at age 21 was still hiding a dating relationship from her foster parents. “I wasn’t allowed to date. And I’m scared to not follow the rules. I did have crushes and I did want to have a boyfriend, but I wanted to follow the rules.” Participants felt that the lack of freedom in foster care held them back from developing social skills and contributed to social anxiety. “I guess you could say [foster care] holds you back from who you are in a way. So we develop our social skills really, really later on in life” (David, cisgender man, gay, 28).

Some participants reacted to the system's control by rebelling against any authority, running away with friends and dating partners, and deciding to abruptly exit the system at age 18. “I wanted out because I wanted my freedom. And when I signed myself out, it was like, ‘What freedom did I end up getting?’” (Madyson, cisgender woman, bisexual, 25). In fact, she found herself in the streets and in abusive relationships. Koral (cisgender woman, straight, 23) echoed her sentiment, both the struggle against the system and the regrets of not staying in extended care and benefiting from available support. “The foster care system had hurt me so much, and I didn't trust anything the system had to offer. If I had done extended care, instead of running away with friends who were doing drugs, I would have gone to college, not gotten pregnant, and avoided a lot of misery down the road.”

Moreover, as Jonathan (cisgender man, gay, 30) explained, the foster care system itself can become a model for a controlling relationship that increases vulnerability for abusive intimate relationships. “Just the feeling of my foster parents always controlling me and the foster care system always controlling me, I needed those [partners] that told me what to do. So, I was very drawn to people that were commanding. So, my relationships were very toxic.”

#### Lack of Connection—“No One Ever Sat Down With Me”

The majority of participants never had conversations with their birth parents about puberty, dating, or sexual health, nor did they have such conversations with foster caregivers and child welfare professionals. “I’ve met some foster caregivers that are like, ‘You’re not my real kid so therefore I don’t have to tell you anything’”(Jasmine, cisgender woman, straight, 21). Some participants inferred that child welfare professionals and caregivers probably thought that “it was too late” for conversations and education about healthy relationships because of the extent of the abuse, specifically sexual abuse, they had experienced in early adolescence. Participants generally were scared to talk about dating and sexual relationships with caregivers or child welfare professionals because they were afraid of potential negative consequences, such as getting kicked out or “having a record” indicating they were dating or sexually active.

Most importantly, without conversations, young people had no opportunities for learning about healthy relationships and caregivers were in the dark about what was going on in the youths’ life and relationships. In the absence of conversations there was no connection or trust between youth and caregivers; “bad decisions and behaviors” became the expression of the youths’ underlying struggles and the focus of the caregiver's response.

Yeah, they [foster parents] didn't know what was going on with me. I guess they figured it was a boy or something for missing out of class and running away a lot. But I'm not sure if they really knew. I don't remember ever talking about it. (Biatriz, cisgender woman, bisexual, 21)

Nobody really knew, they weren’t listening to me. The only way that my foster people knew what was going on was whenever I would make bad decisions. Never once did they sit down and say, ‘Hey, are you okay? Is there something that you need to talk about?’ I felt like I was honestly all alone. (Jessica, cisgender woman, straight, 21)

Some participants like Izzy (cisgender woman, straight, 27) acknowledged that when they were younger it was hard to listen and to process advice about relationships. “I don’t know if people told me and I just didn’t hear them, you know? I was so caught up in my own preconceptions of how people work that I didn’t listen to people when they did try to give me some sort of advice.” Josie reflected on conversations with her long-term mentor after aging out of care. She emphasized that it took years before she was able to absorb the conversations about healthy and unhealthy relationships, open herself up to new perspectives, and make actual changes.

She [mentor] would talk to me about red flags. She would try to show me this is what's healthy, this is what's not healthy. It was hard hearing this for the first time when I'm 18 or 19. My reaction was, ‘This is dumb.’ As I got older, she kept telling me the same things, and I was starting to realize, she's right. But it definitely took a very, very long time—seven or eight years—before I was in this [healthy] relationship. (Josie, cisgender woman, lesbian, 30)

Among all participants there was consensus that they wanted to have conversations with trusted adults where they could process what was going on in their lives. “A lot of kids, even though they don’t ask for it, but deep down inside, they wanna have somebody to talk to” (Marcus, cisgender man, bisexual, 22). Participants also expressed that more than information and advice, they wanted support and the opportunity to learn through experience. “I feel like growing up I got a lot of advice. I don’t feel any of that ever helped me. You really need somebody who cares about you and is going to try and get you to that [healthy] place” (Maya, cisgender woman, bisexual, 23).

#### Stigma—“We’re Going to Be Just Bad People”

Another significant challenge to supportive conversations were the youths’ perceptions that caregivers and child welfare professionals were focusing on their mistakes and assumed they would act like their parents and turn out to be “bad people” (Madyson, cisgender woman, bisexual, 25). “They thought lashing-out behavior was because they’re becoming their father or they must be just trying to break all the rules. It never was, ‘I wonder if they’re struggling emotionally with sexuality and independence?’” (Jonathan, cisgender man, gay, 30). Participants recounted instances where the caregiver's or child welfare professional's fear or presumption that youth were going to repeat the cycle of violence resulted in deep misunderstanding and the youth feeling wrongly “accused.” For example, Koral (cisgender woman, straight, 23) remembered a caseworker who “actually accused me of prostituting myself because I kept running away,” although she was not sexually active and running away had other roots and meaning. Other participants stated that the fact that others expected the worst of them added to their feelings of worthlessness and rebellion against the system, thereby escalating the very behaviors the adults were trying to prevent.

I didn’t care anymore if I got in trouble because they [kinship care providers] were already accusing me of doing it, and it was a combination of just not really having any guidance because they never talked to me about it, and then my boyfriend was two years older than me. He was 16. So, I also felt pressured. I lost my virginity. (Carolina, cisgender woman, straight, 24)

Taken together, these reflections demonstrated how negative assumptions, judgment, and a focus on risk behaviors undermined the youth's sense of self-worth and connections with caregivers. In contrast, participants remembered people who believed in them and their worth, their strength, and ability to make changes. Nancy (cisgender woman, straight, 27) recounted this episode in a group home. “They always thought I didn’t listen, but I really did. I remember her saying don’t ever settle. Think of yourself highly. I’m like, thank you. I listened to it.”

#### Suppression—“My Identity Was Stomped Out”

Participants who identified as lesbian, gay, bisexual, and transgender faced particular challenges with exploring and expressing their identities and experienced a lack of support and outright rejection in placements. Josie entered care at age 15.

I had a normal, low level of care foster home. This is around the time where I started to dress very masculine. And so I had found myself. And I started to date more, and I had a relationship with my roommate—an intimate relationship with one of the other foster girls there. And my foster parent found out and kicked me out for it. (Josie, cisgender woman, lesbian, 30)

Being kicked out started a string of running away episodes, behavioral challenges, and intensified struggles with authority. “So, how am I ever supposed to learn what a healthy relationship looks like if I'm not being taught anything, I'm just being told no or being kicked out?” For Josie and other participants, the experience of rejection by the system followed the experience of rejection in their birth families. More generally, participants noted the absence of any talk about sexual identities, and the absence of positive role models and adequate placements. “There was never any teaching about like, ‘This is what sexual identity is. This is what sexual expression is. You can act one way on the outside but feel something on the inside. The foster parents themselves had no idea how to talk about relationships, talk about identities” (Jonathan, cisgender man, gay, 30). Jonathan concluded, “The system pushed me down to not be who I wanted to be. My identity was dismissed and stomped out.”

### Forming Intimate Relationships

When participants described the history of their dating and sexual relationships, we noted three subgroups: participants that bounced from one abusive relationship to another, participants that had supportive and lasting intimate relationships that they depicted as their chosen family, and participants who avoided intimate relationships.

#### Bouncing From One Abusive Relationship to Another—“I Never Had an Example of What a Healthy Relationship Looks Like”

A majority of participants (*n *= 18) reported involvement in “toxic, ugly, unhealthy, or abusive” relationships that entailed emotional abuse and control, physical and sexual violence, stalking, and sexual exploitation, and that began in adolescence and escalated once they left foster care. Participants attributed their experiences of IPV to not having models for healthy relationships, lacking connections with supportive adults and peers, fears of abandonment, and a low sense of self-worth.

It was a pervasive sentiment among participants that they had never seen and experienced healthy relationships, neither in their birth families nor in foster care. Family members had told them that “everybody is dysfunctional; everybody yells and hits their kids; everybody has mental disorders, and drug abuse, and alcohol abuse” (Koral, cisgender woman, straight, 23).

I never had an example of what a healthy relationship looks like, even with my uncle and his wife [kinship caregivers]. They were always fighting, and she was very controlling and abusive. My mom herself never provided me with a healthy example of a relationship. Her relationship was full of drug abuse and physical abuse. (Carolina, cisgender woman, straight, 24)

Caroline first experienced dating violence in 10^th^ grade. “I kind of was like, ‘Oh, well, somebody wants me, so I'm just going to be with him.’ Now I can say confidently I have abandonment issues, and I wanted to hold onto him even though I knew that he wasn’t good for me.” Like other participants who bounced from one abusive relationship to another, her low sense of self-worth was further eroded by continuing abuse. “I just felt like he drug me through the dirt and I lost a sense of self.”

Given their sense of disconnectedness from birth families and the foster care system, fears of aloneness and abandonment were acute and any relationship, even an unhealthy and abusive relationship, functioned as a “safety blanket.” Homelessness was a common experience (*n *= 8) both in the context of running away and exiting the foster care system and a time of increased vulnerability for victimization. “When I left CPS, I was in several abusive relationships—emotionally, physically, sexually, all of it. I learned how to deal drugs and—the majority of [partners] were off the streets. In the streets, it's not love. It's like a safety blanket” (Madyson, cisgender woman, bisexual, 25).

#### Lasting Intimate Relationships—“Choosing My Family”

A small group of participants (*n *= 5) described lasting and supportive intimate relationships as young adults and did not report experiencing any IPV. Participants in this subgroup all described having positive models for relationships, lasting connections, and a positive sense of self. It was notable that this small group had stable placements in foster care which allowed them to maintain connections at school and in the community, with peers, and with members of their birth families. For example, Miguel (cisgender man, straight, 22) endured years of abuse while living with his aunt, but his girlfriend's home provided respite. He knew early on through experience and observation what a safe and supportive relationship should look like. “I felt safe in this house. I felt wanted as well. And when I’d go home, I could feel the shift of the atmosphere. There was a difference in between how they treated her and then how I was treated whenever I’d go home.” Eventually, Miguel was removed from his aunt's house and requested to be placed with his girlfriend's, now his wife's, family. The strength of their relationship and acceptance in his chosen family continued to anchor his life. “I’m really lucky and fortunate to be able to have such a stable relationship with a person who understands me and enjoys my company. And it’s really a best friend rather than just a girlfriend or a date.”

#### Avoiding Intimate Relationships—“I Just Immediately Start Looking for Red Flags”

Another small group of four participants stated that they had never been in a “solid” relationship, although they occasionally went on dates. They all shared experiences of profound isolation in adolescence resulting from abusive and controlling environments and lacked social experiences with people their own age.

I think my mother raised me in her fears, which is why I was sheltered. But that also disabled me to be social. I didn’t have social skills. I just feel so insecure. I've had to train myself to continue to just socialize and talk to people. Because interacting with people is what's healing me. (Taneka, cisgender woman, straight, 24)

Developing social skills required conscious effort, often with little support from foster caregivers and child welfare professionals. Angelina described consciously observing and “mimicking” social behaviors to integrate with peers.

[In foster care] I was continuously told, ‘You’re socially awkward. You don’t know how to act in social situations. You’re unlovable.’ So, what I tried to do is I would copy everyone else around me, copy what people said. Education was everything to me. I made learning how to interact with people an extra course for me. (Angelina, cisgender woman, straight, 21)

The participants’ self-perceived lack of social skills, anxiety in social situations, and heightened awareness of warning signs of abuse were reflected in their avoidance of intimate relationships as young adults.

I haven’t been in a solid relationship with anybody ever. I think I’m pretty aware of relationships and what's healthy and what's not healthy in a relationship. I may be overly cautious and so I don’t jump into relationships or I try to avoid them. If I date somebody, I just immediately start looking for red flags. (Angelina, cisgender woman, straight, 21)

### Learning and Growth in Emerging Adulthood

At the time of the interviews, participants who had experienced emotionally, physically and/ or sexually abusive dating relationships reported being in a better place, although some were “still rocking the boat” (Madyson, cisgender woman, bisexual, 25) and wondering whether the perceived positive change and healthier relationships might last. This section of the analysis focuses on their process of growth and change as young adults. Participants described how they searched for models of healthy relationships; severed ties with people they considered “toxic” to make room for new connections; and learned to love themselves.

#### Learning Through Trial and Error—“Stepping Outside the Comfort Zone”

Participants concurred that it took time to not only recognize that relationships that seemed “normal” were in fact “abusive,” but then to step outside “the comfort zone.” Several participants expressed that choosing supportive and respectful partners was “scary” or “felt foreign and uncomfortable” because they weren’t used to being treated like that.

You kinda shy away from [healthy relationships] because it's not your comfort zone. When I picked these men, they talked to me like trash and treated me like trash because that's what I was used to and that's what I allowed. That's your comfort zone. And if that's what you’ve been through since you were a child, then that's definitely a really hard habit to break, very hard to break. (Izzy, cisgender woman, straight, 27)

Learning required time and most of all experience—trial and error. While Koral wished to tell other youth that “it can really be better. That they don’t have to settle for someone that treats them like their family did,” she also pointed out that simply being told that relationships could be different would not be enough. Koral described needing to see it and feel it to know that a healthy relationship could be real.

If someone had told me that, I probably would have been like, what’s better? There's no better. If you never are treated well, then you never know what that looks like. It seems like a fantasy, like a fairy-tale. It took a lot of trials with bad relationships for me to figure out how it's supposed to look. And he's really nice to me, and he's really good to me. It's just so different. (Koral, cisgender woman, straight, 23)

#### Making Space for Healthy Connections—“Severing Unhealthy Relationships Even When It Is Painful”

The participants described that ending unhealthy, toxic, and abusive relationships meant that they had to confront fears of abandonment and aloneness. Although necessary, breaking the cycle of abuse often felt “lonely as hell” (Danta, transgender woman, bisexual, 19), especially for participants who had few connections with family, peers, or other caring adults.

I still struggle with all the bad memories of my family and all that abuse. I’ve learned to recognize when people are toxic. I’ve had good practice with having to cut out people that I truly loved. So, that helped me to become strong and be able to sever ties, even when it’s painful. It's lonely, but once I’ve taken those toxic people of out of my life, I’ve started making actual healthy connections. I have a good relationship now. (Koral, cisgender woman, straight, 23)

Although many participants had been offered therapy while in foster care, actively seeking out support and resources as young adults had a different meaning as they took responsibility and had a clear goal in mind. Carolina (cisgender woman, straight, 24) had the following advice for other youth, “I just would hope that nobody would be like me to where they stay in the relationship for too long because they’re scared to be alone or they have those feelings of abandonment. The biggest take-away from all of this, it's always okay to talk to other people and to look for your resources.”

#### Building Self-Worth—“Learning to Love Yourself”

A majority of participants expressed that a sense of unworthiness was so deeply ingrained that it took them a long time, and the support of caring and loving people, to shift their sense of self. Nadia (cisgender woman, bisexual, 23) described how her sense of being “ruined” resulting from severe sexual abuse continued to disrupt placements and relationships and left her homeless. “I was already ruined, so I didn’t want anybody to love me or get that close. So anytime, I would go to any foster home I’d run away. At that time in my life, I didn’t love myself. I didn’t care about myself. I didn’t feel normal and I didn’t deserve that type of love.”

When participants reflected on their learning and growth as young adults, they noted a significant shift in their sense of self. They were “learning to love themselves” and most participants pointed out people in their lives who told them, “You are beautiful, don’t settle.”

Jasmine made the connection between loving and valuing herself, understanding that she could choose partners, and being able to engage in healthy and respectful relationships.

I had no self-esteem. I hated life. I hated myself. I just looked for anyone to just be there and love on me because I just wanted that kind of attention. My advice would be like, learn to love yourself and then—once you learn what you love about yourself—then if you meet a guy that doesn’t like what you love about yourself, then it's not worth the time to just be in that relationship. (Jasmine, cisgender woman, straight, 21)

## Discussion

The present study centered the perspectives of young adults who had lived in foster care during their adolescence. We sought to understand how the unique environment and experience of being in foster care impacted what they learned about relationships, how they formed intimate relationships, and how they described ongoing personal growth as young adults.

In the first section of the analysis, we examined what participants had learned about relationships while in foster care. Our findings revealed missed opportunities to promote healthy relationships. From the perspective of the youth, foster care modelled controlling or disconnected relationships, and did not provide them with working models for healthy relationships. There was a consensus among participants that being in foster care limited opportunities for developing friendships, dating relationships, and exploring their identities. Restrictive placement rules, meant to promote safety, did not allow for normal social activities and development (Pokempner et al., 2015). Furthermore, youth responded to the controlling environment either with silent conformity or rebellion, such as running away and abrupt exit from foster care, which increased risk for IPV as youth experienced instability and homelessness and some engaged in survival sex.

Particularly striking was the widespread absence of conversations with caregivers about dating and sexuality. These findings confirmed previous studies with caregivers and child welfare professionals that revealed discomfort with such conversations and barriers including a lack of training and guidance, confusion about their role and responsibilities, and conflicting values with the youth in their care (Albertson et al., 2018; Harmon-Darrow et al., 2020; Serrano et al., 2018). Our study provided an in-depth exploration of the youth perspective and showed that the lack of conversations was both a symptom and a source of disconnectedness. Youth felt abandoned without guidance and support and expressed themselves behaviorally, whereas caregivers reacted to “acting out” behaviors and “bad choices,” only furthering distrust, disconnect, and placement disruptions. Participants who identified as LGBTQ experienced the lack of conversation and responsiveness on the part of their caregivers as rejection and suppression of their identities. Moreover, conversations and education, when they occurred, largely focused on warning signs of abuse, risks, and presumed negative outcomes which augmented already existing stigma and negative self-concept. Not only did the lack of open and non-judgmental conversations represent a missed opportunity for ongoing, informal education about healthy relationships, but the youth-caregiver disconnect itself contributed to vulnerability for IPV (Katz et al., 2020; Potter & Font, 2019).

Our study also highlighted fragile connections with peers, both as a result of neglect and abuse in the biological family and limitations to normalcy in foster care. A majority of participants in this study perceived themselves as missing social skills or experienced anxiety around people their own age. The role of peer relationships for the development of romantic relationships has been discussed in developmental research (Furman & Rose, 2015), affirming that competencies acquired in peer relationships provide a foundation for reciprocity, intimacy, and validation of self-worth and carry over into romantic relationships. Our study suggests the need to examine peer relationships of youth in foster care both as risk and protective factor for IPV and focus of intervention.

In the second section of the analysis, we examined how youth formed intimate relationships. A majority of participants (67%) reported experiencing IPV at least at one point in their lives, most commonly after leaving foster care, thus confirming other studies that have reported elevated rates of IPV among youth and alumni of foster care (Herrman et al., 2016; Katz et al., 2020). Our study also highlighted the experiences of alumni of foster care who did not report any IPV: Whereas one subgroup enjoyed supportive and lasting intimate relationships that anchored their lives, another subgroup avoided intimate relationships altogether, driven by social anxiety and a cautious focus on warning signs of potential abuse. These findings indicate that research on intimate relationships of youth in foster care needs to capture more nuanced life trajectories and relationship patterns rather than focusing merely on the presence or absence of IPV.

The third section of analysis demonstrated the participants’ resilience and highlighted how they were continuing to develop a sense of self and identity and healthier relationships as young adults. Understanding how youth describe their process of learning and growth can help identify strategies for prevention and intervention that are tailored to their needs and strengths. First, breaking the cycle of violence and finding models for healthy relationships involved trial and error. Information and advice, albeit important, were clearly not enough. Participants needed to experience a supportive, safe, and trusting relationship in order to believe in it; they needed to practice skills for healthy relationships in therapy, with friends, mentors, and caregivers. Above all, breaking patterns of abusive relationships took time—years even—and ongoing support. Second, breaking the cycle of violence also meant bearing loneliness upon ending unhealthy, abusive relationships. Strong connections to a support system were therefore needed to bolster the process. The decision to end toxic or abusive relationships and take steps to confront trauma, mental health needs, and substance use ultimately came from the youth themselves, not from well-meaning and protective, yet controlling, caregivers and professionals. And lastly, participants described building or re-building a positive sense of self and self-worth that allowed them to set expectations in relationships and feel free to deliberately choose a partner. LGBTQ participants expressed the need for an affirmative environment and for having caregivers that provide a safe space to explore their identities and relationships.

### Limitations

Findings of this research study are specific to our convenience sample of young adults with lived experience in foster care: Cisgender women and Hispanic youth were overrepresented, as were youth who emancipated from the foster care system without attaining legal permanency, such as adoption, reunification, or legal guardianship. Future research should find avenues to engage male and transgender youth to gain a deeper understanding of their experience in dating and sexual relationships. Another group that was not represented in our research, were Native American youth who are disproportionately placed in the foster care system. Second, this study focused primarily on the experiences of youth while they were in foster care. We did not include a detailed analysis of abuse and neglect prior to entering foster care or differentiate by the type, time, and duration of the abuse. While it is a strength of our study to highlight youths’ experiences in the foster care environment, we cannot make causal attributions between their experiences in foster care and IPV outcomes. Third, our findings highlight and are limited to the youth perspective and their perception of learning about relationships while in foster care. Listening carefully to the voices of youth in foster care, a hard to reach and vulnerable population, is essential for improving the system and services, yet future studies would be strengthened by a simultaneous exploration of youth and caregiver experiences.

### Implications for Practice and Future Research

This study demonstrates that current practices and programs in foster care are not effective in preventing IPV among youth and young adults. The high prevalence of IPV and associated long-term consequences call for the development of comprehensive prevention and intervention strategies that increase protective experiences in adolescence and help youth build the foundation for healthy relationships with adults, peers, and ultimately with intimate partners. Based on our analysis we suggest that strategies for IPV prevention and intervention need to: provide opportunities for exploration and learning from mistakes; model healthy relationships and increase connections; and affirm youths’ sense of self, self-worth, and identities.

#### Opportunities for Exploration and Learning: Redefining Normalcy

Normalcy, the ability of a child in foster care to live as normal a life as possible and engage in social activities based on their developmental age and behavioral capacity, requires balancing opportunities for exploration and learning with managing risks. Our study showed that all too often this balance is shifted toward safety and control thus limiting adolescents’ developmental needs and freedom to explore friendships and intimate relationships, make decisions, and learn from their mistakes (Pokempner et al., 2015; Simmons-Horton, 2017). Caregivers and child welfare professionals operate based on acute awareness of risks rather than opportunities for learning. Youth will make mistakes, they may indeed become involved in unhealthy friendships and relationships, and they need adults to help them problem solve to develop models and skills for healthier relationships. Restricting social life only increases vulnerabilities and intensifies the questions and problems youth face when they are leaving the system. Promoting healthy relationships in foster care requires shifting from a focus on risks and potential negative outcomes toward focusing on opportunities for learning.

#### Modeling Healthy Relationships and Building Connections: Training for Caregivers

Support for social and emotional development requires intensive training for foster caregivers and child welfare professionals and skill-building programs for youth. While there is consensus that foster caregivers should be encouraged to have conversations about healthy relationships, identity, and sexuality, there is little guidance and training on how to create a safe space for exploring these sensitive topics and maintaining open communication and connection, especially when a youth is exhibiting signs that they may be experiencing dating abuse or be the person perpetrating abuse. Along with training for caregivers, multi-session, inclusive, and skill-building interventions for youth are needed to provide foundational relationship and sexuality education. Multi-level interventions that encompass training and support for child welfare professionals, foster caregivers and mentors, and trauma-informed curricula for youth (Ball et al., 2023; Colarossi et al., 2019) are promising approaches to improving outcomes for youth. Ultimately the relationship between foster caregiver and youth should provide the space to practice skills for healthy relationships that are based on respect, communication, trust, boundaries, and honesty and affirm identity development and self-worth.

#### Affirming Self-Worth and Identity: Destigmatizing Sexuality

Throughout this study we noted that youth experienced stigma, for example when the foster care system expressed negative expectations, focused on their “mistakes,” highlighted risks in sexuality and relationship education, or suppressed sexual identities. Stigmatization in foster care compounded poor self-worth resulting from earlier abuse and neglect. We also noted the tremendous resilience among youth who never tired of trying to make relationships work and continued to learn and work towards healthier, happier, and supportive relationships. Therefore, prevention and intervention programs need to be intentional in avoiding fear- or shame-based based messages around sexuality and relationships, acknowledge lived experience, and affirm a positive sense of self and self-worth.
